# Effects of acute hypoxia on auditory pathway of Wistar albino rats

**DOI:** 10.3906/sag-1911-166

**Published:** 2020-10-22

**Authors:** Nevreste Didem SONBAY YILMAZ, Cem SAKA, Sibel ALİCURA TOKGÖZ, Murat ÇALIŞKAN, Erkan VURALKAN, Ömer BEŞALTI

**Affiliations:** 1 Department of Ear, Nose, and Throat, Antalya Education and Training Hospital, Antalya Turkey; 2 Department of Ear, Nose, and Throat, Ankara Dışkapı Yıldırım Beyazıt Education and Training Hospital, Ankara Turkey; 3 Department of Surgery, Faculty of Veterinary Medicine, Ankara University, Ankara Turkey; 4 Department of Ear, Nose, and Throat, Medical Park Trabzon Hospital, Trabzon Turkey

**Keywords:** Hypoxia, DPOAE, hearing loss

## Abstract

**Background/aim:**

Ischemia is insufficient blood flow to provide adequate oxygenation. In the present study, we aimed to show whether acute hypoxia has a critical oxygen value that may lead to the deterioration of cochlear function.

**Materials and methods:**

Under general anesthesia, prehypoxic signal-to-noise ratios were determined by distortion product otoacoustic emissions (DPOAE). The oxygen saturation (SaO2) values ​​of rats were monitored with an oxygen saturation probe. Rats were injected with an extra dose of anesthetic agent, and SaO2 was reduced. DPOAE values ​​in SaO2 100–90, 90–80, 80–70, and 70–60 posthypoxic values​ were measured and compared statistically with prehypoxic values.

**Results:**

At 3000 and 4000 Hz, SaO2 70–60 values measured after the hypoxia were observed to be statistically significantly lower than the values measured before the hypoxia. At 6000 and 8000 Hz, SaO2 80–70 and 70–60 values measured after the hypoxia were observed to be statistically significantly lower than the values measured before the hypoxia. At 10,000 Hz, all of the values measured after the hypoxia were observed to be statistically significantly lower than the values obtained before the hypoxia.

**Conclusion:**

Many studies have been conducted on the effects of hypoxia on the inner ear. It remains unclear how fluctuations in DPOAE levels affect hearing in clinical trials when the SaO2 starts to decrease. Although hypoxia has been implicated in the etiology of sudden hearing loss and tinnitus, the effects of acute hypoxia on the cochlea are still uncertain. Further studies are needed on this subject.

## 1. Introduction

Ischemia is insufficient blood flow to provide adequate oxygenation that leads to tissue hypoxia or anoxia. Ischemia always results in hypoxia; however, hypoxia can occur without ischemia [1,2]. 

The labyrinthine artery is a terminal artery for the cochlear blood supply. The cochlea is highly dependent on the blood and oxygen supply to maintain its function [1,3]. Therefore, it is very sensitive to hypoxia. However, the tolerance of cochlear cells to ischemia varies between cell types. Afferent and efferent neurons of the cochlear nerve are more susceptible to hypoxia than hair cells [3]. Studies have shown a decrease in distortion product otoacoustic emissions (DPOAE) in ischemia attacks lasting 10 min or less, but these were found to be transient, and they disappeared after the reperfusion period. Ischemic attacks, which last 15–30 min, cause hair cell loss and permanent cochlear dysfunction [3]. The most significant effect of this hypoxia affects hearing high frequencies [4,5]. Reduced cochlear circulation is considered a reason for hearing impairment, such as sudden sensorineural hearing loss or tinnitus [2,4].

The reduction of hearing sensitivity under acute hypoxia has been attributed to the metabolic sensitivity of various electrochemical potentials in the inner ear [1,6]. These potentials can be formed by metabolically active Na/K ions, and a decrease in oxygen is thought to slow down this process. On the other hand, in chronic hypoxia, hearing may improve with the increase in the number of ion pumps in response to the decrease in oxygen [7].

The destructive effect of hypoxia on the cochlea occurs by the reactive products of oxygen formed during the reperfusion period. Reactive oxygen products formed in the event of average oxidative stress can be compensated for by an increase in the synthesis of the antioxidant defense system [1]. In the case of severe oxidative stress, cochlear damage is inevitable because of cell death and the destruction of carbohydrates, proteins, lipids, and nucleic acids [1,7]. However, discussions continue on the critical oxygen level for this severe oxidative stress.

We aimed to show whether acute hypoxia has a critical oxygen value that may lead to the deterioration of cochlear function in a rat study.

## 2. Materials and methods

This study was conducted in Ankara University Faculty of Veterinary Medicine Laboratory of Experimental Animals after approval of Ankara University Animal Experiments Local Ethics Committee dated 24.02.2010 and numbered 201057-287. Sixty-month-old Wistar albino rats weighing 250–300 g were included in the study. These rats were housed in steel cages for 12 h in light and 12 h in darkness at an average temperature of 25 °C. This study complies with International Helsinki Declaration principles.

After anesthesia and examination of the rats, they were transferred to a quiet room. DPOAE was applied using an EchoLab OAE (İstanbul, Turkey) device Labat software. Each rat was positioned horizontally on the floor, and measurements were taken from the right ear. The DPOAE measurements were taken in General Diagnostic mode (2fl-f2 cubic distortion product components). In the measurements, the f2 and f1 frequency ratio (f2/f1) was defined as 1.22, the L1 stimulus severity for f1 frequency and the L2 frequency for f2 frequency, and the measurements were completed at the level of L1 = 65, L2 = 55. After determining that the stimulus wave form was appropriate to the probe indication of the device, the measurements were started. In the evaluation of the DPOAE results, f2 values were selected as 3000, 4000, 6000, 8000 and 10,000 Hz, and the signal/noise ratios at these frequencies were taken for evaluation. 

Statistical analysis was made using IBM SPSS Statistics for Windows, version 23.0 (IBM Corp., Armonk, NY). The normality assumptions were controlled by the the Shapiro–Wilk test. Paired t-test was used for parametric comparison and Wilcoxon signed-rank test was used for nonparametric comparison of measurements before and after the hypoxia. Results are expressed as mean ± SD and median (min–max). P values <0.05 were considered statistically significant. 

All rats included in the test protocol were weighed. For anesthesia, 50 mg/kg ketamine and 6 mg/kg xylazine were given intraperitoneally. The efficiency of anesthesia was controlled by checking the absence of tail, foot, and ear reflexes. Prehypoxic signal-to-noise ratios were determined by DPOAE. The oxygen saturation (SaO2) values of rats were monitored with an oxygen saturation probe. Subsequently, rats were injected with an extra dose of 6 mg/kg xylazine to ensure superficial respiration. No additional action was taken to reduce SaO2. Respiration was superficialized with an anesthetic agent, and the saturation was reduced. The measurement was taken when the SaO2 was 95 mmHg. Four more measurements were made until the SaO2 reached 90 mmHg. The arithmetic mean for each frequency of these measurements was taken and recorded as SaO2 100–90 mmHg. Afterwards, three measurements were taken until the SaO2 decreased from 90 mmHg to 80 mmHg, two measurements were taken between 80 mmHg and 70 mmHg, and the arithmetic mean for these measurements was recorded as SaO2 90–80 mmHg and SaO2 80–70 mmHg. One measurement was taken between 70 mmHg and 60 mmHg, and this value was recorded as SaO2 70–60 mmHg. Ten Wistar albino rats were included in the study. Four animals were excluded from the study because their saturation dropped very quickly during the test, and the SaO2 values ​​could not be obtained from each group. The study was restarted with new 4 Wistar albino rats and the test protocol. Since saturation did not decrease in three animals during the test, an additional dose of 6 mg/kg xylazine was given. Posthypoxic values were planned to be reoxygenated after the test, but after acute hypoxia, seven animals were excluded from the study because the SaO2 could not be increased to 100 mmHg. 

## 3. Results

When we compare the prehypoxic and posthypoxic values ​​of 10 Wistar albino rats, the changes occuring in each frequency of DPOAE according to SaO2 values ​​are as follows.

SaO2 values measured at 3000 Hz before and after the hypoxia are given in Table 1. No statistically significant difference was found between SaO2 values measured before the hypoxia and SaO2 100–90, SaO2 90–80, and SaO2 80–70 values measured after the hypoxia. SaO2 70–60 values measured after the hypoxia were observed to be statistically significantly lower than the values measured before the hypoxia.

**Table 1:** Comparisons between pre- and posthypoxia DPOAE at 3000 Hz. (Wilcoxon signed-rank test.)

	Prehypoxia 3000 Hz (n:10)		Posthypoxia 3000 Hz (n:10)		
	Mean ± SD	Median (min–max)	Mean ± SD	Median (min–max)	P
SaO2 100–90	10.14 ± 18.57	14.81 (–27.33 to 27.48)	16.53 ± 16.39	21.06 (–10.8 to 38.5)	0.139
SaO2 90–80			9.11 ± 15.3	14.64 (–22.8 to 24.72)	0.721
SaO2 80–70			5.4 ± 11.97	7.52 (–15.9 to 23.51)	0.114
SaO2 70–60			–2.69 ± 12.08	0.2 6(–22.8 to 12.29)	0.013

SaO2 values measured at 4000 Hz before and after the hypoxia are given in Table 2. No statistically significant difference was found between SaO2 values measured before the hypoxia and SaO2 100–90, SaO2 90–80, and SaO2 80–70 values measured after the hypoxia. SaO2 70–60 values measured after the hypoxia were observed to be statistically significantly lower than the values measured before the hypoxia.

SaO2 values measured at 6000 Hz before and after the hypoxia are given in Table 3. SaO2 100–90 values measured after the hypoxia were observed to be statistically significantly higher, and SaO2 80–70 and 70–60 values measured after the hypoxia were observed to be statistically significantly lower than the values measured before the hypoxia.

SaO2 values measured at 8000 Hz before and after the hypoxia are given in Table 4. SaO2 100–90 values measured after the hypoxia were observed to be statistically significantly higher, and SaO2 80–70 and 70–60 values measured after the hypoxia were observed to be statistically significantly lower than the values measured before the hypoxia.

SaO2 values measured at 10,000 Hz before and after the hypoxia are given in Table 5. SaO2 100–90, 90–80, 80–70, and 70–60 values measured after the hypoxia were observed to be statistically significantly lower than the values obtained before the hypoxia.

**Table 2:** Comparisons between pre and post-hypoxia DPOAE at 4000 Hz. (Wilcoxon signed-rank test, paired t-test.)

	Prehypoxia 4000 Hz (n:10)		Posthypoxia 4000 Hz (n:10)		
	Mean ± SD	Median (min–max)	Mean±SD	Median (min–max)	P
SaO2 100–90	22.27 ± 8.24	19.58(12.71–34.39)	18.36 ± 6.62	17.36 (11.47–35.16)	0.241
SaO2 90–80			17.69 ± 6.46	16.53 (10.14–29.26)	0.148
SaO2 80–70			16.04 ± 6.15	14.67 (7.16–26.59)	0.073
SaO2 70–60			14.69 ± 6.26	13.11 (8.59–31.06)	0.009

**Table 3:** Comparisons between pre- and posthypoxia DPOAE at 6000 Hz. (Wilcoxon signed-rank test.)

	Prehypoxia 6000 Hz (n:10)		PostHypoxia 6000 Hz (n:10)		
	Mean ± SD	Median (min–max)	Mean ± SD	Median (min–max)	P
SaO2 100–90	12.95 ± 11.15	14.97 (–16.8 to 21.53)	19.24 ± 6.95	19.36 (7.8–33.8)	0.017
SaO2 90–80			12.62 ± 9.79	14.96 (–12.5 to 19.51)	0.721
SaO2 80–70			8.83 ± 6.96	10.71 (–10.5 to 12.82)	0.037
SaO2 70–60			5.47 ± 9.99	8.47 (–21.7 to 11.72)	0.005

**Table 4:** Comparisons between pre- and posthypoxia DPOAE at 8000 Hz. (Wilcoxon signed-rank test.)

	Prehypoxia 8000 Hz (n:10)		Posthypoxia 8000 Hz (n:10)		
	Mean ± SD	Median (min–max)	Mean ± SD	Median (min–max)	P
SaO2 100–90	8.66 ± 22	14.42 (–27.33 to 39.9)	16.76 ± 17.02	17.6 (–4.53 to 43.5)	0.021
SaO2 90–80			11.3 ± 17.37	12.91 (–19.66 to 38.93)	0.721
SaO2 80–70			–4.6 ± 14.87	–5.66 (–28.9 to 12.62)	0.037
SaO2 70–60			–17.57 ± 17.77	–22.92 (–38.41 to 7.23)	0.005

**Table 5:** Comparisons between pre- and posthypoxia DPOAE at 10,000 Hz. (Paired t-test.)

	Prehypoxia 10,000 Hz (n:10)		Posthypoxia 10,000 Hz (n:10)		
	Mean ± SD	Median (min–max)	Mean ± SD	Median (min–max)	P
SaO2 100–90	31.09 ± 8.08	27.84 (20.91–42.87)	22.53 ± 7.02	24.39 (14.19–32.36)	0.011
SaO2 90–80			24.25 ± 4.5	23.11 (18.36–30.75)	0.032
SaO2 80–70			22.88 ± 4.1	22.59 (16.48–31.5)	0.013
SaO2 70–60			23.19 ± 6.15	21.63 (16.06–34.31)	0.040

## 4. Discussion

The ability of the inner ear to function depends on the source of cochlear oxygen; thus, any decrease in oxygen will also result in a decrease in cochlear sensitivity [8,9]). It is thought that reactive oxygen products, which are produced during ischemic reperfusion periods, cause cochlear damage. In addition, the magnitude of this damage depends on the length and severity of the ischemic period [1,10,11].

Rebillard and Lavigne-Rebillard [9] created an artificial hypoxic environment by letting guinea pigs breathe 10% oxygen, and they showed a decrease in DPOAE levels in the hypoxic environment. The DPOAE values did not deteriorate in a mildly hypoxic environment suggesting that the cochlea had a SaO2 value in which the cochlea was operating normally [12,13]. Therefore, the cochlea has an autoregulatory mechanism that provides adequate blood flow in case of mild hypoxia. 

In the present study, we managed to drop the SaO2 level very rapidly. No change in the DPOAE was observed until the SaO2 decreased below 70 mmHg at 3000 Hz and 4000 Hz. This is because, in our opinion, no change was detected in the DPOAE due to the high-level energy supply required by the stria vascularis. However, a statistically significant decrease in the DPOAE was observed as the high-energy depot in the striae vascularis was exhausted when it was below 70 mmHg.

At 6000 Hz, 8000 Hz and 10,000 Hz, there was a statistically significant increase in DPOAE values between 100 and 90 mmHg in the SaO2. We attributed this to the compensation of hypoxia by increased cochlear blood flow [1,4]. Statistically significant decreases were found in the SaO2 80–70 mmHg and 70–60 mmHg at 6000 Hz, 8000 Hz, and 10,000 Hz. There was no statistically significant difference in the SaO2 90–80 mmHg at 6000 Hz and 8000 Hz, while a statistically significant decrease was found at 10,000 Hz. The SaO2 at 10,000 Hz is between 90 and 100 mmHg, i.e. in the case of mild hypoxia, a decrease in DPOAE levels was detected as saturation gradually decreased, even if DPOAE values were elevated. However, this decrease in 6000 Hz and 8000 Hz occurred in the ranges of SaO2 70–60 mmHg and 80–70 mmHg.

These results demonstrate that the cochlea is more susceptible to hypoxia at high frequencies, and there is a change in the DPOAE, even in mild hypoxia, where the SaO2 drops within minutes. Hypoxia is already known to cause hearing loss at high frequencies. If we could look at 12,000 Hz in our study, we could show that these values are falling even further. Our investigation indicates that there is no linear correlation between different oxygen saturation levels. However, more comprehensive studies are needed to have an idea on this issue. 

After cochlear ischemia, Billet [10] showed early ultrastructural changes, such as swelling of outer hair cells, and deterioration of inner hair cells and afferent fibers. Billet stated that these changes occurred earlier than those at the apex of the basal cochlea and blamed the increase in reactive oxygen products. Tabuchi [4] reported that early ultrastructural changes recovered in the reoxygenation period in hypoxia lasting less than 10 min, but hypoxia lasting more than 15 min caused permanent damage to the cochlea. Since this was a preliminary study in which we investigated the effect of acute hypoxia on the cochlea, we did not examine the cochlea ultrastructurally.

Olzowy [5] created a hypoxic environment by exposing nine guinea pigs to a nitric oxide–oxygen mixture. Subsequently, a significant decrease in the DPOAE occurred when the oxygen saturation by reoxygenation returned to the prehypoxic value. The DPOAE gradually increased after reaching the lowest value and reached prehypoxic DPOAE values. In other words, the decrease in the DPOAE occurred when cochlear blood flow started. They attributed this effect to the reactive oxygen radicals during oxygenation.

In their study of 16 healthy male patients, Krisser [13] reduced the level of oxygen in the room from 21% to 13%, and he increased the oxygen value to 21% after forming an 8-h hypoxic environment. They reduced the SaO2 to 78% in the hypoxic interval and found fluctuations in DPOAE values. Even after reoxygenation, there was a significant decrease in the DPOAE level (posthypoxic effect). Therefore, when the SaO2 went below 60, a situation that is incompatible with life occurred. When reoxygenated after regression of SaO2 to 60, the reflexes in rats were not included in the study. Therefore, we could not measure the posthypoxic effect in the present study.

In conclusion, many studies have been conducted on the effects of hypoxia on the inner ear. It remains unclear how fluctuations in DPOAE levels affect hearing in clinical trials when the SaO2 starts to decrease. Although hypoxia has been implicated in the etiology of sudden hearing loss and tinnitus, the effects of acute hypoxia on the cochlea are still uncertain. Further studies are needed on this subject.
